# Synergistic Antibacterial Activity of Combined Antimicrobials and the Clinical Outcome of Patients With Carbapenemase-Producing *Acinetobacter baumannii* Infection

**DOI:** 10.3389/fmicb.2020.541423

**Published:** 2020-10-15

**Authors:** Junyan Qu, Rujia Yu, Qujue Wang, Chunlu Feng, Xiaoju Lv

**Affiliations:** ^1^Center of Infectious Diseases, West China Hospital, Sichuan University, Chengdu, China; ^2^Department of Infectious Diseases, Renshou County People’s Hospital, Renshou, China

**Keywords:** carbapenem-resistant *Acinetobacter baumannii*, infection, resistance mechanism, synergistic effect, clinical outcome

## Abstract

This study aimed to explore the activity of combined antimicrobials *in vitro*, and the relationship among resistance mechanisms, antimicrobial regimens, and the clinical outcome of patients with carbapenem-resistant *Acinetobacter baumannii* (CRAB) infections in western China. A total of 89 CRAB strains were collected from patients with CRAB infection from January 2018 to June 2018. The checkerboard assay was used to study the combined effects *in vitro*. Carbapenemase-encoding genes were detected by polymerase chain reaction (PCR) or multiplex PCR technique. The clinical data of 86 patients were collected. CRAB showed high susceptibility to tigecycline (91.01% inhibition) and polymyxin (83.15% inhibition). Polymyxin plus sulbactam exhibited the highest synergistic effect at a rate of 82.35%. Production of carbapenemase (*bla*_OXA–23_) was the main resistance mechanism of CRAB to carbapenem (95.35%). Excessive expression of active efflux pump genes (*adeB*, *adeJ*, and *abeM*) and deletion of the CarO protein accounted for 13.95% (12/86) and 84.88% (73/86), respectively. The synergistic effect of the sulbactam-based combination was higher than that of the polymyxin B-tigecycline combination for carbapenemase-producing CRAB (*P* < 0.05). The clinical outcome was not affected by the resistance mechanisms (*P* > 0.05). Advanced age, multiple organ dysfunction syndromes (MODS), and admission to the intensive care unit (ICU) were associated with treatment failure (*P* < 0.05). Appropriate antibiotic therapy did not improve the clinical outcome of critically ill patients. Higher minimum inhibitory concentrations (MICs) of tigecycline were associated with treatment failure (*P* < 0.05). A multivariate analysis showed that ICU stay (OR = 15.123, 95% CI: 2.600–87.951, *P* = 0.002) and procalcitonin ≥2 ng/ml (OR = 2.636, 95% CI: 1.173–5.924, *P* = 0.019) were the risk factors for treatment failure. In conclusion, this study demonstrated that the sulbactam-based combination exhibited a synergistic effect *in vitro*. The clinical outcome of patients was not associated with resistance mechanisms. This indicates that the early control of the progression from infection to severe disease may be important.

## Introduction

Carbapenem-resistant *Acinetobacter baumannii* (CRAB) is at the top of the World Health Organization’s list of drug-resistant bacteria and poses a significant threat to human health (Willyard, 2017). It can cause severe infections including pneumonia, bloodstream infection, abdominal infection, and skin and soft tissue infection, for which almost no treatment is available (Willyard, 2017). Data from the China Antimicrobial Surveillance Network (CHINET) has shown that the percentage of imipenem-resistant *Acinetobacter baumannii* (AB) increased from 39.0% in 2005 to 78.1% in 2018 (CHINET, 2019). Infections caused by CRAB have high mortality due to limited antimicrobial options, and CRAB bloodstream infection tends to occur in critically ill patients along with rapid disease progression and higher mortality. A previous study showed that the 30-day mortality of patients with CRAB bacteremia in the intensive care unit (ICU) was as high as 79.8% (Kim et al., 2012). The new β-lactamase inhibitor ceftazidime/avibactam presents an opportunity to improve the survival of patients with carbapenem-resistant *Pseudomonas aeruginosa* and *Enterobacteriaceae* infections, but the susceptibility of CRAB to ceftazidime/avibactam was poor (Hsueh et al., 2019). Several new antimicrobial agents, including aztreonam/avibactam, cefiderocol, and eravacycline, might be promising therapeutic options in treating CRAB infections (Leone et al., 2019). Currently, the treatment options for CRAB infections are limited, including colistin, tigecycline, sulbactam, and aminoglycosides (Karaiskos et al., 2017). Therefore, knowing which of the existing antimicrobials to select in controlling CRAB infections is a huge challenge for clinicians. Observational studies have shown no difference in statistics between monotherapy and combination therapy in mortality and clinical efficacy in patients with CRAB infections (Karaiskos et al., 2017). There are also no convincing studies that recommend the combination of carbapenems, colistin, or sulbactam (Karaiskos et al., 2017).

The resistance mechanisms of CRAB to carbapenem mainly include the production of carbapenemases (especially class D carbapenemases) in AB, excessive expression of an active efflux pump, functional loss of outer-membrane porins (Brink, 2019). Different antimicrobial agents have different antimicrobial mechanisms and targets. Thus, understanding the antibiotic resistance mechanisms exhibited by CRAB will help clinicians to choose the antimicrobial regimens appropriately. At present, some studies have focused on the resistance mechanisms of clinical strains (Jiang et al., 2019; Li S. et al., 2019), and other studies have focused on observational research of the clinical efficacy of different antimicrobial regimens in the treatment of CRAB infections (Elsayed et al., 2019; Russo et al., 2019). Few clinical studies have compared the efficacy of the existing antimicrobial regimens based on different resistance mechanisms. Therefore, it is extremely important to know how to better select from the existing antimicrobials and optimize the treatment regimen according to the antibiotic resistance mechanisms to achieve a better clinical outcome.

This study explored the following three aspects: (1) The *in vitro* activities of imipenem (IPM), biapenem (BIP), sulbactam (SUL), tigecycline (TGC), or polymyxin B (PB), and the combination of IPM-TGC, IPM-BIP, PB-TGC, TGC-SUL, PB-SUL against CRAB were determined. (2) It investigated the mechanisms of carbapenem resistance in clinical CRAB strains. (3) we evaluated the relationship between resistance mechanisms, antimicrobial regimens, and clinical outcomes in patients with CRAB infections.

## Materials and Methods

### Bacterial Strains and Drugs

For this study, eighty-nine non-repeated clinical CRAB strains were collected from patients with CRAB infection between January 2018 and June 2018 in West China Hospital, Sichuan University (a 4300-bed academic tertiary-care hospital). We conducted identification and antimicrobial susceptibility tests (AST) on the VITEK-2 COMPACT automated microbiology system (BioMerieux, France). Species identification was confirmed by detecting and sequencing the *gyrB* and *rpoB* genes. The following drugs were used in these experiments: imipenem/cilastatin sodium (Merck Sharp & Dohme Corp., New Jersey, United States), biapenem (Chia Tai Tianqing Pharmaceutical Co., Ltd., Jiangsu, China), cefoperazone/sulbactam sodium (Pfizer, New York, United States), polymyxin B (SPH NO.1 Biochemical & Pharmaceutical Co., Ltd., Shanghai, China), tigecycline (Wyeth Pharmaceutical Co., Ltd., Carolina, United States), and carbonyl cyano-p-chlorphenizone (CCCP) (Sigma-Aldrich, Inc., United States).

### Clinical Isolates and Antimicrobial Susceptibility Testing, and Synergistic Testing and Determination of Efflux Pump Activity

The minimum inhibitory concentrations (MICs) of IPM, BIP, TGC, SUL, and PB were determined using the broth microdilution method following CLSI guidelines (CLSI, 2017). The susceptibility breakpoints of the antimicrobials used against *A. baumannii* in this study were in accordance with the CLSI criterion (CLSI, 2017). The breakpoint for BIP was defined as ≥ 8 mg/L resistant strains based on the breakpoints for other carbapenems against *A. baumannii*, because of the lack of CLSI, European Committee on Antimicrobial Susceptibility Testing (EUCAST), or FDA-approved breakpoint for BIP. The breakpoints for TGC were based on the FDA-approved tigecycline breakpoints for the Enterobacteriaceae, ≤2 mg/L susceptible and ≥ 8 mg/L resistant. *Escherichia coli* ATCC 25922 was used as the reference strain. The synergistic effects of antimicrobial combinations were determined using the checkerboard method with Mueller-Hinton broth (Gordon et al., 2010). The fractional inhibitory concentration indices (FICI) were calculated for each of the paired antimicrobials using the following equation: the MIC of drug A in combination/the MIC of drug A alone + the MIC of drug B in combination/the MIC of drug B alone. FICI<1, FICI = 1.0, and 1<FICI< 2.0 were used to define synergy, addition, and indifference, respectively, according to a previous study (Yoon et al., 2004). MICs of IPM were observed in the absence and the presence of CCCP (10 μg/mL). A significant inhibition effect was defined as a four-fold or more reduction in IPM MICs in the presence of CCCP. Efflux pump activity was determined for the efflux pump positive isolates.

### Polymerase Chain Reaction (PCR) and Nucleotide Sequencing

Classes A and D carbapenemase-encoding genes (*bla*_GES_, *bla*_KPC_, *bla*_OXA–51_, *bla*_OXA–23_, *bla*_OXA–24_, *bla*_OXA–58_, and *bla*_OXA–143_) and class B metallo-β-lactamase genes (*bla*_IMP_, *bla*_VIM_, *bla*_SPM_, *bla*_GIM_, *bla*_SIM_, and *bla*_NDM_) were detected by the PCR method or multiplex PCR technique as previously described (Poirel et al., 2000; Woodford et al., 2006; Ellington et al., 2007; Higgins et al., 2010). The primers were synthesized and the amplicons obtained were sequenced by the Beijing Genomics Institute. The sequences were analyzed with the NCBI BLAST program^1^.

### Fluorescence Quantitative Real-Time RT-PCR

The expression levels of efflux system genes (*adeB*, *adeJ*, and *abeM*) were assessed using fluorescence quantitative real-time reverse transcription-PCR (qRT-PCR). The *adeB* gene was screened as described previously (Peleg et al., 2007) and new primers were designed for the *adeJ* (*adeJ*-F: ATGAGAAA CTGATTGCAGCTC; *adeJ*-R: TGAGGAGTATCTTCCTGAC CA) and *abeM* (*abeM*-F: AGGCTTCGGCTTATCGAAAC; *abeM*-R: AGAGGGCTAAGGACCAATGC) genes.

The RNA was extracted using TaKaRa RNAiso Plus (TaKaRa Bio Inc., Japan), and cDNA was synthesized using the PrimeScript RT reagent Kit with gDNA Eraser (TaKaRa Bio Inc., Japan). Real-time PCR assays were performed using an Option 2 real-time PCR detection system (Bio-Rad Inc., United States) with the SsoFast EvaGreen Supermix Kit (Bio-Rad Inc., United States). The 16S rRNA gene was used as the internal control gene. Expression analysis was performed using the 2^–ΔΔCt^ method (Livak and Schmittgen, 2001) to compare the relative expression of the mRNA with that of *A. baumannii* ATCC 19606.

### Western Blot Analysis

Western blot analysis was used to evaluate the level of the outer membrane protein CarO expression for each isolate. Bacterial cells were mixed with radioimmunoprecipitation (RIPA) buffer (3 ml RIPA buffer/g). After centrifugation, the supernatant was collected. Western blotting was performed as previously described (Goh et al., 2013). The CarO-specific antibody was generated in a rabbit by Abmart Biomedicine Co., Ltd. (Shanghai, China; 1: 1000 dilution). The secondary antibody was horseradish peroxidase (HRP) conjugated goat anti-rabbit IgG (Fumaisi Biotechnology Co., Ltd., Nanjing, China; 1: 2000 dilution).

### Clinical Data

Detailed clinical information of patients with CRAB infection, including demographic characteristics, laboratory data, antibiotic treatment regimens, and clinical outcomes, were collected from their medical records. The clinical and microbiological diagnosis of CRAB infection was defined according to the criteria of the Centers for Disease Control and Prevention (CDC) (Horan et al., 2008). Colonization was excluded before the beginning of this study. CRAB was defined as *A. baumannii* demonstrating resistance or intermediate susceptibility to one or more of meropenem or IPM, according to the AST results. Appropriate antibiotic treatment was defined as the use of at least one active drug against CRAB within 5 days of the date of sampling. Treatment outcomes were evaluated at 28 days after clinical samples were taken from patients. Clinical outcomes were defined as treatment success or treatment failure (death or disease progression). This study was approved by the Ethics Committee of West China Hospital, Sichuan University.

### Statistical Analysis

Statistical analyses were performed using SPSS version 22.0 for Windows (SPSS Inc., Chicago, United States). Normality tests for quantitative variables were assessed by the Shapiro-Wilk test. Significance testing was performed using the Student’s *t* test or Mann-Whitney U test. Categorical variables were compared using the Chi-square test or Fisher’s exact test. Risk factors for 28-day treatment outcomes of patients with CRAB infection were identified using a logistic regression model. All variables with *P* < 0.05 in univariate analysis were entered into the multivariate analysis by using the forward conditional method. *P* < 0.05 (two-tailed) was considered statistically significant.

## Results

### *In vitro* Antibacterial Activity of Antimicrobials Alone and in Combination

The sensitivity rates of CRAB to IPM, BIP, SUL, TGC, and colistin were 0, 0, 2.25% (2/89), 91.01% (81/89), and 83.15% (74/89), respectively. Some strains were selected to evaluate the drug interaction in antibiotic combinations according to the MIC values (excluding highly susceptible and resistant strains). MICs of antimicrobials against CRAB alone and in combination are shown in Table 1. For all drug combinations, a synergistic effect was the most common interaction. PB-SUL showed the highest synergistic effect at a rate of 82.35% (14/17), followed by TGC-SUL at a rate of 73.91% (17/23).

**TABLE 1:** Minimum inhibitory concentration (MIC) determination of antimicrobials against carbapenem-resistant *A. baumannii* isolates.

Single antibiotic, MIC (mg/L)					Antibiotic combination, MIC (mg/L)								

	Range	MIC_50_	MIC_90_	R (n, %)		Range	MIC_50_	MIC_90_	R (n, %)	FICI (Median, IQR)	Synergism (n, %)	Additive (n, %)	Indifference (n, %)
*N* = 24						Imipenem + Tigecycline							
Imipenem	16–256	64	64	24(100)	Imipenem	4–256	16	64	21 (87.5)	0.75 (0.59–0.75)	14 (58.33)	2 (8.33)	8 (33.33)
Tigecycline	0.25–4	1	2	0 (0)	Tigecycline	0.06–1	0.5	1	0 (0)				
*N* = 24						Imipenem + Biapenem							
Imipenem	16–256	64	64	24 (100)	Imipenem	2–128	16	32	18 (75)	0.69 (0.63–0.75)	16 (66.67)	6 (25.0)	2 (8.33)
Biapenem	8–128	32	128	24 (100)	Biapenem	2–64	16	64	19 (79.17)				
*N* = 17						Polymyxin B + Tigecycline							
Polymyxin B	0.5–4	1	2	1 (5.88)	Polymyxin B	0.06–2	0.5	1	0 (0)	0.75 (0.50–0.75)	7 (41.18)	3 (17.65)	7 (41.18)
Tigecycline	0.5–2	1	2	0 (0)	Tigecycline	0.03–1	0.25	1	0 (0)				
*N* = 23						Tigecycline + Sulbactam							
Tigecycline	0.25–2	1	2	0 (0)	Tigecycline	0.0075–1	0.125	0.5	0 (0)	0.50 (0.63–0.75)	17 (73.91)	5 (21.74)	1 (4.35)
Sulbactam	32–512	64	128	19 (82.61)	Sulbactam	8–256	32	64	6 (26.09)				
*N* = 17						Polymyxin B + Sulbactam							
Polymyxin B	0.5–4	1	2	1 (5.88)	Polymyxin B	0.125–1	0.25	1	0 (0)	0.75 (0.55–0.75)	14 (82.35)	2 (11.76)	1 (5.88)
Sulbactam	32–256	64	256	15 (88.24)	Sulbactam	8–128	32	128	6 (35.29)				

*R, resistance; MIC, minimum inhibitory concentration; FICI, fractional inhibitory concentration Index; IQR, interquartile ranges.*

### Association of the Clinical Outcome and Resistance Mechanisms

We collected the clinical data of 86 patients with CRAB infections. Three patients were lost because, by the time the diagnosis was made, they were transferred to other hospitals. The distribution of carbapenem resistance mechanisms in these clinical isolates is shown in Table 2. The only detectable carbapenemase gene was *bla*_OXA–23_ (82, 95.35%). All strains detected the naturally occurring *bla*_OXA–51_ gene (86, 100.00%). The sequences of OXA51 and OXA23 obtained in this study have been submitted to GenBank and the accession numbers are MT757793 and MT757794, respectively. Other carbapenemase-encoding genes were not detected. The MIC values of 12 isolates were decreased at least four-fold in the presence of CCCP, and they were also the active efflux phenotype positive strains. The relative expression of *adeB*, *adeJ*, and *abeM* had increased by approximately 4.4-fold, 4.1-fold, and 3.8-fold, respectively, in CRAB isolates than in the sensitive strain. The positive rate of CarO porin in the treatment success group was higher than that in the treatment failure group (*P* < 0.05).

**TABLE 2:** Distribution of carbapenem resistance mechanisms in clinical isolates of carbapenem-resistant *Acinetobacter baumannii*.

	All (*N* = 86) (%)	Treatment success (*N* = 62) (%)	Treatment failure (*N* = 24) (%)	*P*-value
**Carbapenemase-encoding genes**				
Class D oxacillinases				
*bla*_OXA–23_	82 (95.35)	59 (95.16)	23 (95.83)	1.000
**Drug efflux pumps**				
Efflux pump inhibition test (+)	12 (13.95)	8 (12.90)	4 (16.67)	0.731
*AdeB* (fold) (Mean ± SD)	4.37 ± 2.99	4.88 ± 3.44	3.35 ± 1.74	0.544
*AdeJ* (fold) (Mean ± SD)	4.14 ± 2.52	4.71 ± 2.82	2.99 ± 1.45	0.432
*AbeM* (fold) (Mean ± SD)	3.80 ± 2.09	4.41 ± 2.30	2.57 ± 0.88	0.336
**Porin**				
CarO	73(84.88)	58(93.55)	15(62.50)	**0.002**

*SD, standard deviation. These bold values emphasize that the difference is statistically significant.*

### The *in vitro* Antibacterial Activity of Antimicrobial Combination in CRAB With Different Resistance Mechanisms

The synergistic effect of PB in combination with SUL was higher than that of the other drug combinations against CRAB strains (*P* < 0.05). In the strains with carbapenemases, the synergistic effect of the SUB-based combination was higher than that of PB-TGC combination (*P*<0.05). For other resistance mechanisms, no drug combination showed a statistically significant difference in the synergistic effect (*P* > 0.05) (see Table 3).

**TABLE 3:** The synergistic effect of drug combinations in carbapenem-resistant *Acinetobacter baumannii* with different resistance mechanisms.

Antibiotic combination	Synergism (n, %)	Resistance mechanisms		
		Carbapenemases	Drug efflux pumps	Functional loss of CarO
Imipenem + Tigecycline (*N* = 24)	14 (58.33)	12 (50.00)	2 (8.33)	2 (8.33)
Imipenem + Biapenem (*N* = 24)	16 (66.67)	14 (58.33)	4 (16.67)	1 (4.17)
Polymyxin B + Tigecycline (*N* = 17)	7 (41.18)^a^	6 (35.29)^c^	2 (11.76)	1 (11.76)
Tigecycline + Sulbactam (*N* = 23)	17 (73.91)^b^	16 (69.57)^d^	4 (17.39)	3 (13.04)
Polymyxin B + Sulbactam (*N* = 17)	14 (82.35)^c^	12 (70.59)^e^	3 (17.65)	3 (17.65)

**P*^*ab*^ < 0.05; *P*^*ac*^ < 0.05; *P*^*cd*^ < 0.05; *P*^*ce*^ < 0.05.*

### Clinical Characteristics of Patients With CRAB Infection

After excluding 4 patients with non-carbapenemase-producing CRAB infection, a total of 82 patients with CRAB infections (mean age 58.05 ± 17.54 years; 51 male) were included in this study (Table 4). The treatment failure rate was 28.05% (23/82) within 28 days of the specimens being taken. Patients at an advanced age, those with multiple organ dysfunction syndromes (MODS), and those admitted in the ICU had a higher rate of treatment failure (*P* < 0.05). Patients in the treatment failure group had higher levels of procalcitonin (PCT) and percentage of neutrophils, and a lower level of estimated glomerular filtration rate (eGFR) than those in the treatment success group (*P* < 0.05). The treatment failure group had a higher proportion of CRAB patients with bloodstream infections than the treatment success group (*P*<0.05). More patients had a poor clinical outcome even if they were given appropriate antibiotic therapy (*P*<0.05). Patients infected with CRAB strains with lower MICs of TGC had a better clinical outcome (*P* = 0.021).

**TABLE 4:** Clinical characteristics in patients with carbapenem-resistant *Acinetobacter baumannii* infection.

Variables	All (*N* = 82) (%)	Treatment success (*N* = 59) (%)	Treatment failure (*N* = 23) (%)	*P*-value
Male (n, %)	51 (62.20)	39 (66.10)	12 (52.17)	0.243
Age (yr) (Mean ± SD)	58.05 ± 17.54	53.66 ± 19.26	63.61 ± 11.65	**0.026**
MODS (n, %)	37 (45.12)	20 (33.90)	17 (73.91)	**0.001**
In ICU when specimen was obtained (n, %)	42 (51.22)	22 (37.29)	20 (86.96)	**0.000**
**Laboratory data**				
PCT (ng/ml), (Median, IQR)	0.63 (0.16–2.15)	0.40 (0.11–1.59)	1.11 (0.68–5.87)	**0.001**
WBC (× 10^9^/L), (mean ± SD)	10.87 ± 5.43	10.20 ± 5.48	12.61 ± 4.97	0.071
Neutrophils (%),(mean ± SD)	83.05 ± 8.97	80.61 ± 10.14	86.07 ± 6.12	**0.001**
Hemoglobin (g/L), (mean ± SD)	95.98 ± 22.60	98.09 ± 21.77	90.57 ± 24.24	0.177
Albumin(g/L), (mean ± SD)	32.12 ± 5.39	32.42 ± 4.39	31.35 ± 7.44	0.522
eGFR (ml/min), (mean ± SD)	84.44 ± 37.21	90.07 ± 37.11	70.23 ± 34.13	**0.030**
**Site of infection (n, %)**				
Lung	53 (64.63)	40 (67.80)	13 (56.52)	0.337
Intra-abdominal	8 (9.76)	5 (8.47)	3 (13.04)	0.680
Blood	10 (12.20)	4 (6.78)	6 (26.09)	**0.026**
Urinary tract	4 (4.88)	4 (6.78)	0 (0)	0.573
Wound	6 (7.32)	6 (10.17)	0 (0)	0.178
Central nervous system	1 (1.22)	0 (0)	1 (4.35)	0.284
**Antibiotic Treatment** (n, %)				
Appropriate antibiotic therapy	52 (63.41)	33 (55.93)	19 (82.61)	**0.024**
Monotherapy	16 (19.51)	11 (18.64)	5 (21.74)	0.048
Combination therapy	36 (43.90)	22 (37.29)	14 (60.87)	
**MIC (mg/L)** (Median, IQR)				
Imipenem	128 (64,128)	128 (64,128)	128 (64,128)	0.325
Biapenem	64 (32,64)	64 (32,64)	64 (64,64)	0.303
Sulbactam	64 (64,128)	64 (64,128)	64 (64,128)	0.693
Polymyxin B	2 (1,2)	2 (1,2)	2 (1,2)	0.181
Tigecycline	1 (1,2)	1 (1,2)	1 (0.5,2)	**0.021**

*MODS, multiple organ dysfunction syndromes; ICU, intensive care unit; PCT, procalcitonin; WBC, white blood cell; eGFR, estimated glomerular filtration rate; MIC, minimum inhibitory concentration; SD, standard deviation; IQR, interquartile ranges. These bold values emphasize that the difference is statistically significant.*

### Risk Factors for a Poor Treatment Outcome of Patients With CRAB Infection

Multivariate analysis showed that ICU stay (OR = 15.123, 95% CI: 2.600–87.951, *P* = 0.002) and PCT ≥ 2 ng/ml (OR = 2.636, 95% CI: 1.173–5.924, *P* = 0.019) were independent risk factors for treatment failure in patients with CRAB infection, as shown in Table 5.

**TABLE 5:** Risk factors associated with treatment failure of patients with carbapenem-resistant *Acinetobacter baumannii* infection.

Variables	*P*-value	Odds ratio(OR)	95% CI
Ages	0.156	1.037	0.986–1.089
MODS	0.114	3.752	0.729–19.320
In ICU when specimen was obtained	**0.002**	15.123	2.600–87.951
PCT (≥2 ng/ml)	**0.019**	2.636	1.173–5.924
Neutrophils (Percent)	0.950	1.034	0.359–2.982
eGFR	0.660	1.163	0.593–2.280
Appropriate antibiotic therapy	0.798	1.248	0.227–6.856
MICs of tigecycline	0.799	0.870	0.296–2.551
Bloodstream infection	0.975	1.030	0.166–6.406

*MODS, multiple organ dysfunction syndromes; ICU, intensive care unit; PCT, procalcitonin; eGFR, estimated glomerular filtration rate; MIC, minimum inhibitory concentration. These bold values emphasize that the difference is statistically significant.*

## Discussion

In this study, we found that CRAB displayed high sensitivity to TGC and PB, which is similar to that reported in many previous reports (He et al., 2015; Yazdansetad et al., 2019). Despite their *in vitro* activity against CRAB, the results of studies on the clinical effectiveness of TGC have been mixed, and even show a higher mortality risk (McGovern et al., 2013). A low serum concentration of TGC is inadequate to eradicate CRAB, and this limits its use in CRAB bloodstream infection. The role of TGC in treating CRAB infections remains controversial. Nephrotoxicity and neurotoxicity of colistin limited its clinical use, and patients with carbapenem-resistant bacterial infections receiving colistin monotherapy had a less than optimal outcome (Zusman et al., 2017). The combination of several antimicrobial agents produced synergistic effects in this study, especially the PB-SUL combination. Although many previous studies have demonstrated the synergistic or additive effects of an antimicrobial combination (He et al., 2015; Le Minh et al., 2015), the antibacterial activity *in vitro* does not necessarily reflect activity *in vivo*. In clinical practice, the efficacy of TGC and polymyxin was uncertain even when used in combination with other antimicrobials (Zusman et al., 2017; Doi, 2019). A previous study showed that the polymyxin-vancomycin combination produced a synergistic effect *in vitro* (Bae et al., 2016). Vancomycin may enhance the permeability of polymyxin on the *A. baumannii* outer membrane, and perhaps other antimicrobial agents, such as ceftazidime, aztreonam, and meropenem, may also have this effect, which can be studied further.

A high percentage of OXA-23-positive isolates were observed in this study. Carbapenemase production was the main resistance mechanism of CRAB to carbapenem. However, different types of resistance mechanisms in CRAB did not affect the clinical outcome. A previous study found that patients infected with *bla*_KPC–2_-producing carbapenem-resistant *Enterobacteriaceae* (CRE) had poor outcomes (Wang et al., 2018). Mutations in *bla*_KPC–3_ in CRE may lead to a decrease in the antibacterial activity of ceftazidime-avibactam (Haidar et al., 2017). Early detection of the carbapenemase type might help improve the prognosis of patients with CRE infections. With respect to CRAB, studies on the relationship between the clinical outcome and specific resistance genes are limited. A previous study showed that mortality in critical patients with *A. baumannii* complex bacteremia was not correlated with any specific genospecies (Lee et al., 2014). Resistance mechanisms might not play a significant role in the clinical outcomes of patients with CRAB infection, but further studies are needed.

For strains with the presence of carbapenemases, more isolates exhibited synergistic activity with a combination of PB/TGC and SUL, but there was no statistically significant difference in the synergistic effect for the strains with overexpression of efflux pump genes and functional loss of porin. Leelasupasri et al. (2018) also found that colistin combined with SUL has a good synergistic and additive effect on CRAB strains, and colistin plus SUL against CRAB seemed to be an interesting option. There are limited studies on the *in vitro* antibacterial activity of an antimicrobial combination based on different resistance mechanisms. An open-label, randomized controlled trial found that colistin-meropenem combination was not superior to colistin monotherapy in the cumulative survival rate (Paul et al., 2018). Although many combinations, such as SUL-TGC, SUL-carbapenems, polymyxin-carbapenems, and polymyxin-TGC, were used for CRAB infection (Chinese XDR Consensus Working Group et al., 2016), the efficacy of colistin-SUL against CRAB in clinical use also has to be evaluated. The 28-day treatment failure rate in these patients was 28.05% (23/82). The patients at an advanced age, those with MODS, those with a lower level of eGFR, and those admitted to the ICU had a poor clinical outcome, which was similar to that in previous studies (Sheng et al., 2010; Niu et al., 2018; Du et al., 2019). The treatment failure rate increased with the advanced age of patients, possibly associated with a decreased immune response to pathogens as age increases (Li M. et al., 2019). MODS, a lower level of eGFR, and ICU stay often indicate that the patients are critically ill and are more likely to die.

This study also showed that the level of PCT in the treatment failure group was significantly higher than that in the treatment success group. PCT has now been considered as a marker for early diagnosis, prognosis, and antibiotic stewardship in patients with bacterial infections, especially acute respiratory infections and sepsis (Lee et al., 2020).

Some previous studies have shown that appropriate therapy could decrease the mortality in patients with CRE bloodstream infections (Gutiérrez-Gutiérrez et al., 2017; Wang et al., 2018). In this study, we found that appropriate antibiotic therapy did not improve the clinical outcome of patients with CRAB infection. Most of the patients in the treatment failure group were in a critical condition when they developed CRAB infection. MODS is associated with very high mortality (Ramírez, 2013), and their clinical outcome was still poor even if they were given aggressive antibiotic therapy. Therefore, early identification of bacterial infection, immediate administration of antibiotic therapy, and prevention of severe infection-induced MODS are very important to reduce bacterial infection-related mortality. This study also found that the group that died had higher TGC MICs. Some patients were given TGC because polymyxin was not available in China at that time. Lower MICs of TGC can more easily meet the pharmacokinetic/pharmacodynamic parameter requirements.

In our study, multivariate analysis showed that ICU stay and PCT ≥ 2 ng/ml were closely correlated with a poor outcome of patients with CRAB infection. Previous studies showed that there were many risk factors for treatment failure in CRAB infection, including ICU stay, TGC therapy, presence of septic shock, higher acute Physiology and Chronic Health Evaluation II (APACHE II) score, central venous catheterization, inappropriate empirical antimicrobial treatment, and multiple organ failure (Sheng et al., 2010; Niu et al., 2018; Du et al., 2019). High PCT values often represent severe infection, which often occurs in critically ill patients and easily leads to death. A previous systematic review found that inappropriate empirical antimicrobial treatment was a risk factor for higher mortality in patients with CRAB infections (Du et al., 2019), which was different from that in our study. Further multicenter, high-quality, prospective studies are needed.

This study has some limitations. First, since the MIC values of TGC and polymyxin of many strains were very low, only some strains were evaluated for the *in vitro* antibacterial activity of an antimicrobial combination. Second, only three patients were treated with polymyxin because it was not available in our hospital at that time. Therefore, clinical data on polymyxin combined with SUL was not available. Relevant studies will be carried out in the future.

## Conclusion

In conclusion, this study demonstrated the synergistic potential of the combination of polymyxin/TGC and SUL *in vitro* against CRAB presenting carbapenemase-encoding genes. The clinical outcome of patients was not associated with resistance mechanisms. The patients at an advanced age, those with MODS, and those admitted to the ICU had a poor clinical outcome. ICU stay and PCT ≥ 2 ng/ml were risk factors for treatment failure in patients with CRAB infection. Appropriate antibiotic therapy did not improve the clinical outcome of critically ill patients. Infections with higher MICs of TGC were more difficult to control. It may be more important to identify the infection early, actively control its progression to severe infection, and reduce ICU admission.

## Data Availability Statement

All datasets generated for this study are included in the article/supplementary material.

## Ethics Statement

The studies involving human participants were reviewed and approved by the Ethics Committee of West China Hospital, Sichuan University. Written informed consent for participation was not required for this study in accordance with the national legislation and the institutional requirements.

## Author Contributions

XL and JQ conceived of and designed the study. XL supervised the experiments. JQ, RY, and CF performed the experiments. QW and CF collected the data. JQ, RY, and QW analyzed or interpreted the data. JQ wrote the draft. All authors read, revised, and approved the final manuscript.

## Conflict of Interest

The authors declare that the research was conducted in the absence of any commercial or financial relationships that could be construed as a potential conflict of interest.

## Footnotes

1 www.ncbi.nlm.nih.gov/blast/
